# SF-Transformer: A Mutual Information-Enhanced Transformer Model with Spot-Forward Parity for Forecasting Long-Term Chinese Stock Index Futures Prices

**DOI:** 10.3390/e26060478

**Published:** 2024-05-30

**Authors:** Weifang Mao, Pin Liu, Jixian Huang

**Affiliations:** 1 Business School, Central South University, Changsha 410083, China; wfmao@hnu.edu.cn; 2 School of Computer and Engineering, Central South University, Changsha 410083, China; jiandanglp@csu.edu.cn; 3 School of Geosciences and Info-Physics, Central South University, Changsha 410083, China

**Keywords:** stock index futures, financial complexity, long-term forecasting, mutual information, transformer

## Abstract

The complexity in stock index futures markets, influenced by the intricate interplay of human behavior, is characterized as nonlinearity and dynamism, contributing to significant uncertainty in long-term price forecasting. While machine learning models have demonstrated their efficacy in stock price forecasting, they rely solely on historical price data, which, given the inherent volatility and dynamic nature of financial markets, are insufficient to address the complexity and uncertainty in long-term forecasting due to the limited connection between historical and forecasting prices. This paper introduces a pioneering approach that integrates financial theory with advanced deep learning methods to enhance predictive accuracy and risk management in China’s stock index futures market. The SF-Transformer model, combining spot-forward parity and the Transformer model, is proposed to improve forecasting accuracy across short and long-term horizons. Formulated upon the arbitrage-free futures pricing model, the spot-forward parity model offers variables such as stock index price, risk-free rate, and stock index dividend yield for forecasting. Our insight is that the mutual information generated by these variables has the potential to significantly reduce uncertainty in long-term forecasting. A case study on predicting major stock index futures prices in China demonstrates the superiority of the SF-Transformer model over models based on LSTM, MLP, and the stock index futures arbitrage-free pricing model, covering both short and long-term forecasting up to 28 days. Unlike existing machine learning models, the Transformer processes entire time series concurrently, leveraging its attention mechanism to discern intricate dependencies and capture long-range relationships, thereby offering a holistic understanding of time series data. An enhancement of mutual information is observed after introducing spot-forward parity in the forecasting. The variation of mutual information and ablation study results highlights the significant contributions of spot-forward parity, particularly to the long-term forecasting. Overall, these findings highlight the SF-Transformer model’s efficacy in leveraging spot-forward parity for reducing uncertainty and advancing robust and comprehensive approaches in long-term stock index futures price forecasting.

## 1. Introduction

Futures contracts, standardized agreements for the delivery of assets at specified prices and times, serve as important hedging tools for investors, including those in the stock market [1]. Stock index futures, based on stock indices, mitigate risks and losses for stock investors while also addressing systematic risks. Despite their relatively recent introduction, stock index futures have gained significant traction globally, emerging as popular derivatives vital for maintaining financial market vitality and gauging capital market maturity [2,3,4,5]. China, recognizing the importance of financial market development and hedging instruments, has diligently cultivated its stock index futures market [6,7,8]. The establishment of the CSI 300 Stock Index Futures in 2010 marked the market’s inception at the China Financial Futures Exchange (CFFEX), followed by expansions to include additional index futures. Notable milestones, such as the relaxation of short-selling restrictions and the introduction of new index futures contracts, have contributed to increased trading volume and market liquidity. This ongoing development, crucial for deepening capital market reforms and bolstering financial market effectiveness, highlights the significance of financial complexity analyses in anticipating market changes and optimizing the price discovery function of China’s stock index futures market [9,10,11,12].

The long-term stock index futures prediction plays a crucial role in advancing China’s stock index futures market [13,14]. This research topic has garnered significant attention. The prevailing approach in this field predominantly relies on financial time series analysis [15,16]. However, due to the inherent complexity and uncertainties of financial derivatives, their prices and related time series often exhibit nonstationary, nonlinear characteristics, deviating from the normal distribution assumptions typically mandated by most financial time series analysis methods [17,18]. To address these limitations, there has been an introduction of machine learning methods that rely on much fewer assumptions regarding time sequences and processes [19,20,21]. The machine learning methods operate in a data-driven fashion, enabling the efficient capture of nonlinear features within the data and enhancing the accuracy of stock index futures prediction.

Deep learning, as an advanced subset within the realm of machine learning methods, has demonstrated remarkable success across various applications [22,23,24,25,26]. Unlike conventional “shallow” machine learning models, which lack distributed representations and necessitate manual feature extraction [27], our method empowers automatic feature extraction from the data [26,28]. Additionally, in deep learning, the hidden layers serve as linear combinations of input features, with the weights between the hidden and input layers mirroring the weights of input features in this linear combination [29]. Moreover, Montufar et al. [30] demonstrated that the capacity of deep learning models grows exponentially with increasing depth. Attributed to these merits, deep learning methods may achieve more promising forecasting results in predictive analyses of stock index futures [31,32,33,34,35,36,37,38,39].

Despite the advancements in methods for predicting stock index futures, these approaches operate purely in a data-driven fashion, emphasizing the principle of letting the data speak for itself [40,41]. However, given the inherent volatility and dynamic nature of financial markets, it remains challenging to robustly forecast future market movements [17,42,43,44]. The Chinese Stock Index Futures market is relatively new, and the introduction of futures for market indices may be premature [45,46]. The stock index futures market in China, with an average daily trading volume of about 100 thousand lots (CSI 300), is characterized by stringent regulations aimed at controlling speculation and encouraging hedging, primarily focusing on domestic enterprises and institutional investors. In contrast, the USA’s stock index futures market [5], featured by a high daily trading volume of approximately 200 million lots (S&P 500), is highly developed, with extensive product offerings and diverse participation from both institutional and retail investors, allowing for significant speculative activities. Meanwhile, Hong Kong’s stock index futures market [47,48], with a daily trading volume of about 100 thousand lots, serves as a vital link between Chinese and global investors, offering high liquidity and transparency with a mix of international and local participation. Therefore, China’s market is still in its early development stage. When forecasting future prices with purely data-driven machine learning techniques, the ever-evolving market regulatory policies, relatively low market transparency, and shorter exchange history contribute to higher financial complexity and uncertainty in forecasting and risk management compared to the more mature and stable futures markets of the USA and Hong Kong. Additionally, enterprise and institutional investors, who are the major participants in Chinese markets, aim for long-term hedging. They are less concerned with short-term fluctuations and more focused on avoiding significant financial losses over the long term. This requirement greatly increases the difficulty of using data-driven machine learning models for predictive analysis and risk management research on stock index futures.

In this paper, we integrate financial theory and deep learning methods. Our idea is inspired by the following observation: while a data-driven machine learning method is adept at allowing the data to speak for itself and forecasting data variation tendencies based on historical patterns, financial theory is more robust in addressing financial uncertainty as it takes into account future variations stemming from a comprehensive understanding of economic factors and market dynamics. Thus, the mutual information generated by financial theory has the potential to significantly reduce uncertainty in long-term forecasting. We leverage financial theory to guide the application of deep learning methods, thereby combining the advantages of these two categories of methods and further improving the predictive accuracy and risk management capabilities for China’s stock index futures market. To this end, we propose SF-Transformer: a mutual information-enhanced Transformer model with spot-forward parity for forecasting long-term Chinese Stock Index Futures prices. Initially, the SF-Transformer model is raised based on the arbitrage-free futures pricing model, i.e., spot-forward parity. We conduct a descriptive analysis on the data required for predicting stock index futures prices in China, evaluating predictions from the short term to the long term and comparing predictions among different stock index futures. A comparative analysis is conducted using different models, including the Transformer model, LSTM, MLP, and the stock index futures arbitrage-free pricing model, highlighting the superiority of the SF-Transformer model in both short-term and long-term predictions of stock index futures. Significantly, increased mutual information highlights the crucial role of spot-forward parity in addressing complexity and uncertainty in long-term forecasting. Based on the research findings, recommendations are presented for governments and businesses.

The primary contributions of this paper are as follows: First, as stated above, we introduce a novel forecasting model integrating spot-forward parity and the Transformer model. This innovative approach notably improves the accuracy of short- and long-term forecasting for the Chinese Stock Index Futures prices. Second, unlike the majority of prior studies that concentrate on analyzing the trend of individual time series stock index futures to make predictions, we identify that the stock index price, the risk-free rate, and the stock index dividend yield play an important role in Chinese Stock Index Futures price forecasting.

The organization of this paper is as follows: Section 2 presents the methodology employed for prediction creation, including the measures and statistical tests utilized to assess forecast accuracy. Section 3 offers a comprehensive overview of the data utilized in this study. Section 4 gives the experimental design, while Section 5 presents the results and discussion pertaining to the forecast of stock index futures prices. Section 6 concludes the paper and proposes avenues for future research.

## 2. Methodology

### 2.1. Spot-Forward Parity Stock Index Futures Pricing Model

Since the inception of stock index futures in 1982, research on pricing methods for stock index futures has been a core topic in both the theory and practice of stock index futures. In traditional microeconomics, the analysis of demand and supply curves is primarily based on the spot market, where transactions occur immediately. However, the completion of forward and futures contracts is delayed, meaning that goods are ordered first, and payment is made at a future date. Currently, the mainstream model for futures pricing is the arbitrage-free trading model, proposed by Cornell and French in 1983. The basic principle is that if a financial derivative can be replicated using existing prices and known financial instruments, the price of the financial derivative must be equal to the price of the replicated financial instruments; otherwise, there is an arbitrage opportunity [49]. The fundamental assumptions that the arbitrage-free pricing theory needs to satisfy are as follows:

**Assumption** **1.** *The market has no friction. In other words, there are no transaction costs in the financial market*.

**Assumption** **2.** *Market participants do not bear counterparty risk*.

**Assumption** **3.** *The market is perfectly competitive*.

**Assumption** **4.** *Market participants are risk-averse and desire more wealth*.

**Assumption** **5.** *There are no arbitrage opportunities in the market. Arbitrage refers to the process in which an investor can obtain a risk-free return without the need for an initial investment in the trading of a certain asset*.

In the presence of the abovementioned assumptions, to price stock index futures, the following two asset portfolios, A and B, can be constructed with their initial positions at time = 0:

Portfolio A: Short position in a forward contract.

Portfolio B: Short position in $e^{-qT}$ units of the underlying spot, and a long position in a zero-coupon risk-free bond with a value of $S\left(0\right)e^{-qT}$.

Here, the risk-free interest rate is denoted as $r_{f}$, q is the stock index dividend yield, and S(0) is the stock index price at the initial time t = 0. Let $\Pi_{A}\left(t\right)$ and $\Pi_{B}\left(t\right)$ represent the values of portfolios A and B at time t. Since the forward contract has no initial cost, and the amount obtained from the short position in the spot is exactly invested in the long position of the zero-coupon risk-free bond, the values of these two portfolios at the initial time t=0 are:

(1) $$\Pi_{A}\left(t\right)=0,$$

(2) $$\Pi_{B}\left(t\right)=0.$$

As the zero-coupon risk-free bond earns a return of $e^{r_{f}T}$, the values of these two portfolios at time t=T are:

(3) $$\Pi_{A}\left(T\right)=F_{T}\left(0\right),$$

(4) $$\Pi_{B}\left(T\right)=S\left(0\right)e^{\left(r_{f}-q\right)T}.$$

Since portfolio B replicates portfolio A, the values of these two portfolios are equal at time t=T, leading to:

(5) $$F_{T}\left(0\right)=S\left(0\right)e^{\left(r_{f}-q\right)T}.$$

When the risk-free interest rate $r_{f}$ does not change over time and remains consistent for all terms, theoretically, the price of the stock index futures equals the forward price with the same delivery date and underlying spot. Therefore, Equation (5) represents the arbitrage-free trading stock index futures pricing model. In empirical applications, researchers often take the logarithm of both sides to linearize it [50,51,52,53,54]:

(6) $$ln\left(F_{T}\left(0\right)\right)=ln\left(S\left(0\right)\right)+\left(r_{f}-q\right)T.$$

From the above equation, it is evident that the price fluctuation of stock index futures is primarily influenced by factors such as the spot price (i.e., stock index price), risk-free interest rate, and stock index dividend yield. The impact of these factors has been extensively confirmed through empirical studies [52,53,55,56,57].

Although the classical arbitrage-free trading stock index futures pricing model is widely used by investors and institutions, it can only provide the theoretical price of the current futures and does not offer meaningful insights into future futures price movements. Furthermore, this arbitrage-free model relies on strict assumptions, which are often not met in real markets. For instance, risk-free interest rates fluctuate in real markets, with longer terms typically resulting in higher prices. Stock index futures trading incurs costs, and investors are often influenced by emotions, which can be irrational. These factors contribute to small deviations of futures market prices from theoretical prices, making it challenging to predict future stock index futures prices accurately based solely on theoretical prices.

Therefore, there is a need to enhance the arbitrage-free pricing model to better guide its role in futures forecasting.

### 2.2. Transformer Neural Network Model

The Transformer model was originally proposed for natural language processing (NLP). After being proposed for several years, the advent of Transformers in the realm of deep learning has marked a paradigm shift, particularly in the context of NLP and various sequential data tasks [58,59,60,61,62]. Traditional sequence models for time sequence forecasting, such as recurrent neural networks (RNNs) [63,64,65] and long short-term memory networks (LSTMs) [66,67,68,69,70], faced challenges in capturing long-range dependencies and suffered from sequential processing inefficiencies. Recently, Transformers have rapidly emerged as the cornerstone of numerous cutting-edge models, owing to their capability to capture intricate patterns in sequential data. Transformers, proposed by Vaswani et al. [71], addressed these limitations by leveraging a novel mechanism called *self-attention*.

Mathematically, for a given input sequence X, the self-attention first computes its Query Q, Key K, and Value V Matrices:

(7) $$Q={XW}_{Q},K={XW}_{K},V={XW}_{V},$$

where X represents the input sequence, and $W_{Q}$, $W_{K}$, and $W_{V}$ are learnable weight matrices. Based on the compatibility between the query and key, attention scores can be computed to represent the importance assigned to each element in the sequence.

(8) $$AttentionScores\left(Q,K\right)=softmax\left(\frac{{QK}^{T}}{\sqrt{d_{k\;}}}\right),$$

where $d_{k}$ is the dimension of the key vectors, which is used to scale the dot product to mitigate issues related to vanishing gradients. The self-attention is the weighted sum that combines the values according to their corresponding attention scores, capturing the contextual information.

(9) $$Attention\left(Q,K,V\right)=AttentionScores\left(Q,K\right)\cdot V.$$

The self-attention mechanism allows each element within the sequence to simultaneously consider all other elements, capturing their contextual relationships in X effectively.

To enrich the expressive capacity of self-attention, Transformers utilize multi-head attention. This involves applying the attention mechanism multiple times in parallel, each with different learned linear projections.

(10) $${Head}_{i}=Attention\left({QW}_{Qi},\;{KW}_{Ki},\;{VW}_{Vi}\right),$$

where $W_{Qi},W_{Ki},$ and $W_{Vi}$ are learnable weight matrices specific to the i-th head. The outputs from these multiple heads are then concatenated and linearly transformed.

(11) $$MultiHead\;Output=Concat\left({Head}_{1},\ldots ,{Head}_{h}\right)W_{O}.$$

The outputs from the individual heads are concatenated and linearly transformed by the matrix $W_{O}$ to produce the final multi-head attention output.

Transformers lack inherent positional information due to the unordered nature of self-attention. For processing sequence data, positional encoding is introduced in Transformers. Given the position pos of an element in the time-sequence data, its positional encoding PosEnc can be calculated as:

(12) $$PosEnc(pos,2i)=sin(\frac{pos}{{10,000}^{\frac{2i}{d}}}),$$

(13) $$PosEnc(pos,2i+1)=cos(\frac{pos}{{10,000}^{\frac{2i}{d}}}),$$

where i is the dimension and d is the model’s hidden dimension. The positional encoding is finally added to the values of the original time points in the sequence data, enabling the self-attention mechanism to process sequence data.

Combining the positional encoding, the multi-head attention forms a network layer in the Transformer model to extract features of sequence data. Based on this foundational layer, the Transformer architecture consists of an encoder responsible for processing the input sequence and a decoder tasked with generating the output sequence. Both encoder and decoder consist of multiple layers, each containing self-attention mechanisms and feedforward neural networks. The encoder transforms the input sequence into a set of continuous representations, and the decoder generates the output sequence step by step, utilizing the encoder’s representations.

The advantages of Transformers are multifaceted. By employing self-attention mechanisms, Transformers excel in capturing long-range dependencies, making them highly efficient for tasks involving sequence data. The parallelized processing of sequences and the ability to consider context holistically contribute to their success in various applications beyond NLP, including computer vision, speech processing, and financial sequence forecasting. For details of Transformers, interested readers are referred to reference [71].

**Figure 1:**
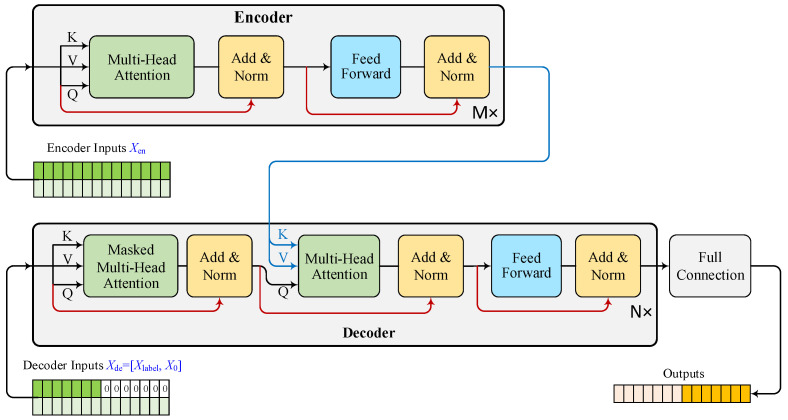
Transformer model for stock index futures price forecasting. The time series data are input into the encoder, which employs multiple attention layers to extract features for forecasting. Simultaneously, the forecasting horizons, marked as zeros and accompanied by the previous historical time series data, are fed into the decoder. The decoder, integrating features from the encoder, predicts the values of forecasting horizons using multiple attention layers and a fully connected layer.

Utilizing the Transformer model, we can forecast the future price by leveraging a given sequence of historical stock index futures data (Figure 1). Considering a historical data sequence as input $X_{en}$, it undergoes encoding and is fed into the Transformer model’s encoder. The encoder extracts inherent features from the historical data sequence and forwards them to the attention layers in the decoders, serving as Keys K and Values V. The decoder receives the input in the form of a decoder sequence $X_{de}$, composed of a subsequence preceding the forecasting sequence within the input sequence to the encoder and the subsequence to be forecast, in which the sequence to forecast is marked by zero values. Taking $X_{de}$ as Values V, the decoder uses multiple attention layers to explore the interconnection between encoded features, i.e., Keys K and Values V, and forecast the target variables, i.e., historical stock index futures prices in our setting. Formally, the output Y of the Transformer model for stock index futures price forecasting can be formulated as:

(14) $$Y=FC\left(Decoder\left(X_{de},Encoder\left(X_{en}\right)\right)\right),$$

where FC(⋅) denotes fully-connected layers.

### 2.3. SF-Transformer Network Model

The spot-forward stock index futures pricing model asserts that the price of stock index futures is predominantly influenced by the stock index price, risk-free interest rate, and stock index dividend yield. This underscores the importance of incorporating these three financial factors into the prediction of future prices. Subsequent empirical studies [45,53,57,72] have consistently demonstrated that these factors, integral to the spot-forward arbitrage-free pricing model, exert a lasting impact on futures prices. On the other hand, given that historical prices of stock index futures represent typical time series data, there is a notable implication that the Transformer model can be effectively used to capture the temporal volatility characteristics inherent in stock index futures. Therefore, integrating the spot-forward futures pricing model and Transformer network allows for the assimilation of financial knowledge from arbitrage-free pricing theory and the temporal volatility characteristics of stock index futures, thereby enhancing the effectiveness of stock index futures price forecasting.

To improve the predictive performance of stock index futures prices, this paper proposes the SF-Transformer, an enhanced Transformer futures prediction model by integrating the spot-forward arbitrage-free futures pricing model. In the SF-Transformer model, the information from the financial market contained in the arbitrage-free trading model and historical stock index futures information are stacked in a common vector. The introduction of this information increases the Transformer cell input from scalar data of stock index futures prices to a 4-dimensional vector. Consequently, the learnable weight matrices $W_{Q}$, $W_{K}$, and $W_{K}$ in Equation (10) are four times larger than that of the original weight matrix. Based on the theoretical estimation of the Vapnik–Chervonenkis dimension in neural network theory [73,74,75,76], the increase in the number of parameters in the Transformer model significantly enhances the model’s capacity, theoretically improving its learning ability to express stock index futures prices.

Given the SF-Transformer illustrated in Figure 1, we can formally show the introduction of the three variables in the spot-forward parity is able to improve the model. Let X represent the sequence data including variables of stock index futures prices $X_{p}$ and variables of spot-forward parity $X_{SF}$, the self-attention operation in Equation (9) is rewritten as:

(15) $$Q={XW}_{Q}=\left(X_{p},X_{SF}\right)\left(\begin{array}{c} W_{Q,p}\\ W_{Q,SF} \end{array}\right)=X_{p}W_{Q,p}+X_{SF}W_{Q,SF}=Q_{p}+Q_{SF},$$

(16) $$K={XW}_{K}=\left(X_{p},X_{SF}\right)\left(\begin{array}{c} W_{K,p}\\ W_{K,SF} \end{array}\right)=X_{p}W_{K,p}+X_{SF}W_{K,SF}=K_{p}+K_{SF},$$

(17) $$V={XW}_{V}=\left(X_{p},X_{SF}\right)\left(\begin{array}{c} W_{V,p}\\ W_{V,SF} \end{array}\right)=X_{p}W_{V,p}+X_{SF}W_{V,SF}=V_{p}+V_{SF},$$

where $W_{\left(\cdot \right),p}$ and $W_{\left(\cdot \right),SF}$ denote the weighting matrices related to $X_{p}$ and $X_{SF}$, respectively, and $\left(Q_{p},K_{p},V_{p}\right)$ and $\left(Q_{SF},K_{SF},V_{SF}\right)$ are the query, key, and value matrices corresponding to the stock index futures prices and the spot-forward parity.

Given the query Q, key K, and value V matrices in Equations (15)–(17), the attention score is rewritten as:

(18) $$\begin{array}{ll} AttentionScore\left(Q,K\right) & =softmax\left(\frac{{QK}^{T}}{\sqrt{d_{k\;}}}\right)=softmax\left(\frac{\left(Q_{p}+Q_{SF}\right){\left(K_{p}+K_{SF}\right)}^{T}}{\sqrt{d_{k\;}}}\right)\\ & =softmax\left(\frac{Q_{p}K_{p}^{T}+Q_{p}K_{SF}^{T}+Q_{SF}K_{p}^{T}+Q_{SF}K_{SF}^{T}}{\sqrt{d_{k\;}}}\right)\\ & =Z^{-1}softmax\left(\frac{Q_{p}K_{p}^{T}}{\sqrt{d_{k\;}}}\right)⊙softmax\left(\frac{Q_{p}K_{SF}^{T}}{\sqrt{d_{k\;}}}\right)⊙softmax\left(\frac{Q_{SF}K_{p}^{T}}{\sqrt{d_{k\;}}}\right)⊙softmax\left(\frac{Q_{SF}K_{SF}^{T}}{\sqrt{d_{k\;}}}\right)\\ & =softmax\left(\frac{Q_{p}K_{p}^{T}}{\sqrt{d_{k\;}}}\right)⊙\left(Z^{-1}softmax\left(\frac{Q_{p}K_{SF}^{T}}{\sqrt{d_{k\;}}}\right)⊙softmax\left(\frac{Q_{SF}K_{p}^{T}}{\sqrt{d_{k\;}}}\right)⊙softmax\left(\frac{Q_{SF}K_{SF}^{T}}{\sqrt{d_{k\;}}}\right)\right)\\ & =softmax\left(\frac{Q_{p}K_{p}^{T}}{\sqrt{d_{k\;}}}\right)⊙S \end{array}$$

where ⊙ denotes the element-wise product, $Z^{-1}$ is the diagonal matrix for normalization, and S denotes the attention score matrix related to $X_{SF}$. Equation (18) indicates that the variables of spot-forward parity can be introduced to adjust attention scores.

Given decomposition of V in Equation (17), the self-attention is rewritten as:

(19) $$\begin{array}{ll} Attention\left(Q,K,V\right) & =AttentionScore\left(Q,K\right)\cdot V\\ & =AttentionScore\left(Q,K\right)\cdot \left(V_{p}+V_{SF}\right)\\ & =AttentionScore\left(Q,K\right)\cdot V_{p}+AttentionScore\left(Q,K\right)\cdot V_{SF}. \end{array}$$

Equation (19) indicates that, after introducing spot-forward parity, self-attention values are adjusted according to the value matrix $V_{SF}$, which encodes the information with respect to financial factors.

**Figure 2:**
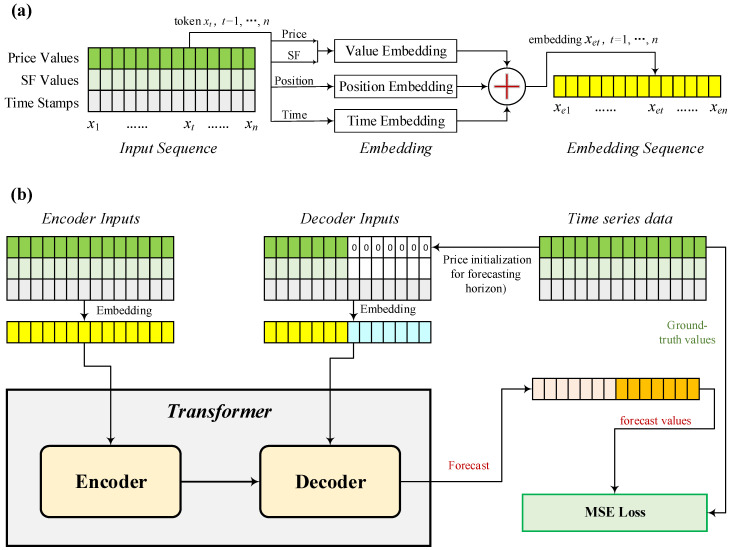
Architecture of SF-Transformer. (**a**) Sequential spot-forward (SF) parity values, stock index futures values, and global time constitute the input representation to the SF-Transformer. This input generates embeddings for SF via value/position/time embedding. (**b**) The SF-Transformer utilizes the embeddings of the encoder and decoder inputs to generate forecasts of stock index futures prices in a generative manner. Model training involves the use of mean squared error (MSE) to measure the difference between forecasted values and ground-truth values.

Based on the above account, our SF-Transformer model is shown in Figure 2. For a time series of stock index futures, we add three variables of spot-forward parity as additional dimensions. And then, we added position encoding defined in Equations (12) and (13) and time stamp encoding representing the global time context (minutes, hours, dates, and holidays). Based on this representation, the encoder processes inputs in the form of long sequence time series. The self-attention operation is employed to extract attentions. On the other hand, the decoder handles the other long sequence time series, in which a subsequence padded with zeros is the target element to be forecast. The decoder generates the weighted attention of the encoded features and promptly generates values of target elements in a generative fashion. For training the SF-Transformer model, the MSE loss on prediction in terms of the target element is used. The loss is backpropagated through the decoder and encoder to update the model parameters.

### 2.4. Forecasting Accuracy Assessment

In this study, the mean absolute percentage error (MAPE), a well-established evaluation criterion [63,77,78,79], is employed to assess the performance of the above models. MAPE serves as a metric for evaluating the accuracy of predictive models, particularly in the context of time series forecasting. It is derived by calculating the absolute percentage difference between the predicted values ${\hat{y}}_{t}$ and the actual values $y_{t}$ for each data point in the dataset, and then taking the average of these differences across all data points. The equation for MAPE is thus:

(20) $$MAPE=\frac{1}{T}\sum_{t}\frac{\left|{\hat{y}}_{t}-y_{t}\right|}{y_{t}}\times 100.$$

Here, T represents the total number of elements in the time series.

Unlike metrics like RMSE, which are squares of the errors, MAPE provides a clear percentage representation of how far off the predictions are, relative to the magnitude of the actual values. This makes it particularly insightful for understanding the relative accuracy of forecasts, especially in scenarios where the scale of the data varies significantly. For instance, in financial markets, MAPE can highlight the average magnitude of forecast errors relative to the actual stock prices, which is crucial for assessing the practical significance of forecasting errors.

### 2.5. Mutual Information Estimation

The long-term fluctuations in stock index futures prices exhibit intricate patterns, presenting formidable obstacles to accurate forecasting. While spot-forward parity models hold theoretical promise in mitigating uncertainty during forecasting, it is essential to substantiate the impact of spot-forward models on stock index futures price prediction. To assess the contribution of spot-forward models, we examine the information gain derived from spot-forward parity. To this end, we employ mutual information as a measure before and after incorporating spot-forward parity. Mutual information, closely tied to the entropy of a random variable, serves as a fundamental metric in information theory. Put simply, mutual information quantifies the level of information or relationship between two variables.

Given two continuous random variables x and y, the mutual information of x and y, denoted as $I\left(X;Y\right)$, is defined as:

(21) $$I\left(x;y\right)=\int p(x,y)\log\left(\frac{p(x,y)}{p\left(x\right)p\left(y\right)}\right)dxdy,$$

where p(x,y) is the joint probability density function of x and y, and $p\left(x\right)$ and $p\left(y\right)$ are the marginal probabilities of x and y, respectively. Mutual information can also be stated as:

(22) $$I\left(x;y\right)=H\left(y\right)-H\left(y|x\right),$$

where $H\left(y\right)$ is the entropy of y and $H\left(y|x\right)$ is the conditional entropy given x.

Using variation of mutual information, the information gain from spot-forward parity $X_{SF}$ can be estimated as:

(23) $$C\left(X_{SF}\right)=I\left(X_{p},X_{SF};Y\right)-I\left(X_{p};Y\right),$$

where Y denotes the stock index futures price. From Equations (22) and (23), we know that the information gain measured by the variation of mutual information, denoted as $C\left(X_{SF}\right),$ is equivalent to the differences of conditional entropy given variables with and without spot-forward parity $X_{SF}$:

(24) $$C\left(X_{SF}\right)=H\left(Y|X_{p}\right)-H\left(Y|X_{p},X_{SF}\right).$$

It is known that a normal distribution achieves maximum entropy among all distributions with the equivalent covariance ([80], Theorem 8.6.5). The entropy of the normal distribution $N\left(\mu ,\sigma \right)$ with mean μ and standard deviation σ is $\frac{1}{2}\log\left(2\pi \sigma^{2}\right)+\frac{1}{2}$. By combining Equation (22), the lower bound of mutual information can be derived, which has been discussed in the literature [81]:

(25) $$I\left(x;y\right)\geq H\left(y\right)-\left(\frac{1}{2}\log\left(2\pi \sigma^{2}\right)+\frac{1}{2}\right).$$

By using the lower bound of mutual information in Equation (25), and eliminating the constant terms, it is straightforward to derive the approximation of $C\left(X_{SF}\right)$ in Equation (23) that measures the variation of mutual information with and without spot-forward parity $X_{SF}$:

(26) $$C\left(X_{SF}\right)\approx \log\sigma_{p}-\log\sigma_{p,SF},$$

where $\sigma_{p}$ and $\sigma_{p,SF}$ are the standard deviations of the normal distribution given $X_{p}$ and $\left(X_{p},X_{SF}\right)$, respectively. Thus, the calculation of $C\left(X_{SF}\right)$ becomes an estimation of standard deviations. Assuming the normal distribution $p\left(Y|X_{p},X_{SF}\right)$ (resp., $p\left(Y|X_{p}\right)$) satisfies $p\left(Y|X_{p},X_{SF}\right)\sim N\left(\mu \left(X_{p},X_{SF}\right),\sigma_{p,SF}\right)$ (resp., $p\left(Y|X_{p}\right)\sim N\left(\mu \left(X_{p}\right),\sigma_{p}\right),$), where $\mu \left(X_{p},X_{SF}\right)$ is the mean given $X_{p}$ and $X_{SF}$, we can estimate $\sigma_{p,SF}$ (resp., $\sigma_{p}$) in a maximum likelihood fashion:

(27) $$\sigma_{p,SF}^{2}=\frac{1}{N}\sum_{i=1}^{N}{\left(Y^{\left(i\right)}-\mu \left(X_{p}^{\left(i\right)},X_{SF}^{\left(i\right)}\right)\right)}^{2}.$$

Here, $X_{p}^{\left(i\right)},X_{SF}^{\left(i\right)}$, and $Y^{\left(i\right)}$ denote i-th known samples of historical prices, spot-forward parity, and forecasted prices. Since the optimal mean $\mu \left(X_{p},X_{SF}\right)$ is theoretically intractable [82], to determine $\mu \left(X_{p},X_{SF}\right)$, we follow the work of [83], using the outputs of the empirically optimal predictor in experiments to approximate $\mu \left(X_{p},X_{SF}\right)$.

## 3. Data

In September 2006, with the approval of the China Securities Regulatory Commission (CSRC), the China Financial Futures Exchange (CFFEX) was established in Shanghai, marking a new milestone in the reform of China’s capital markets. In 2010, CFFEX introduced the country’s first stock index futures financial instrument, the CSI 300 Stock Index Futures (IF). During the bull market in 2015, it also launched the SSE 50 Stock Index Futures (IH) and the CSI 500 Stock Index Futures (IC). Simultaneously, it enriched the variety of investment instruments in China’s financial derivatives market and enhanced the operational efficiency of the financial system.

Futures with stock indices as underlying assets are referred to as stock index futures. Currently, there are four stock index futures financial instruments in China: IF, IH, IC, and IM. IF’s underlying assets are composed of 300 representative stocks listed on the Shanghai Stock Exchange (SSE) and Shenzhen Stock Exchange (SZSE). These sample stocks usually have large market capitalization and strong liquidity. IH’s underlying assets come from the SSE and consist of only 50 stocks, but they are large-cap stocks with excellent liquidity, representing the overall situation of benchmark enterprises in various industries. IC’s underlying assets are also composed of stocks from the SSE and SZSE, totaling 500, with relatively smaller market capitalization, easily influenced by major funds, and often exhibiting larger price fluctuations and higher index volatility. IM’s underlying assets consist of 1000 stocks excluding the sample stocks from the CSI 800 index, characterized by smaller size and good liquidity. However, due to its recent listing of less than six months, the data volume is limited. Therefore, this research selects the daily data of IF, IC, and IH as the research and forecasting objects.

The data used in this research are obtained entirely from the Wind database, primarily including daily closing price data of the main contracts of major stock index futures in China and financial market information data contained in the no-arbitrage futures pricing model. The financial market information includes stock index prices, risk-free interest rates, and stock index dividend yields. Specifically, the daily closing prices of the CSI 300 stock index, CSI 500 stock index, and SSE 50 stock index are selected as stock index prices. Following the conventions of previous studies [53,57], the overnight weighted average interest rate of interbank lending is chosen as the risk-free interest rate. The dividend yield is derived from the dividend yields of the aforementioned stock indices. According to the structure of the SF-Transformer model, the logarithm of the closing prices of stock index futures and stock indices is taken in this section.

To facilitate comparative analyses across different stock index futures, the selected sample data period is uniformly set from 16 April 2015 to 25 October 2022, encompassing 1832 trading days. In previous research, there has been no theoretical consensus on the optimal selection ratio for the training–validation–testing sets [84,85,86,87,88]. The training set ratios generally range from 70% to 90%, with the most common choice being 80% [69,79,89,90,91,92,93,94,95,96,97]. Therefore, based on the tradition of previous research, this study constructs the training, validation, and testing sets in a ratio of 8:1:1. Specifically, data from 16 April 2015 to 20 April 2021 are selected as the training set, data from 21 April 2021 to 18 January 2022 are selected as the validation set, and data from 19 January 2022 to 25 October 2022 are chosen as the testing set.

The results of the descriptive statistical analysis for the data selected in this research are presented in Table 1. It can be observed that most variables exhibit skewness and kurtosis, indicating a departure from the normal distribution assumption required by many financial time series analysis methods. In comparison to the CSI 300 Stock Index Futures and its spot market, as well as the SSE 50 Stock Index Futures and its spot market, the volatility of the CSI 500 Stock Index Futures and its spot market is approximately twice as large. This aligns with the characteristics of the CSI 500 Stock Index Futures and its spot market, as discussed earlier.

## 4. Experiments

### 4.1. Experimental Setting

To validate whether integrating the no-arbitrage futures pricing model with the SF-Transformers can enhance the predictive performance of stock index futures prices, this section conducts experiments on stock index futures price prediction.

While financial regulatory authorities focus on mitigating risks in the futures market through early awareness of derivative market risks, facilitating prompt and comprehensive responses for prevention and mitigation, enterprises and institutions utilizing futures for long-term hedging are less concerned with short-term minor fluctuations. Instead, their primary apprehension lies in the potential for substantial financial losses over an extended period. In comparison to short-term predictions made a day or a few hours later, mid- to long-term predictions are more challenging and often less accurate, as they require the model to capture complex dependencies and robustness against noise in the data over extended periods. Given mid- and long-term forecasting is the major concern for enterprises and institutions, this study investigates various forecasting time horizons (1, 3, 5, 7, 10, 14, 21, and 28 days) and assesses the predictive performance of the models using the MAPE as a metric for forecasting error.

In our experiments, the Transformer model is designed to include encoders and decoders with 2 and 1 attention layers, respectively. We carefully tune these hyperparameters to ensure model performance. We finally use 8 multi-head attention layers. The Adam optimizer [98] was utilized to train the Transformer model. Several hyperparameters play a pivotal role in shaping the predictive performance of the Transformer model during training, including parameters such as batch size, epochs, and learning rate. The early stopping training strategy was implemented to counteract overfitting. Notably, the learning rate, deemed the most critical tunable hyperparameter, was initially set relatively high at 0.01 during training to facilitate a swift reduction in the loss function early on. Subsequently, as the training progressed, the learning rate underwent decay with a rate of 0.5.

### 4.2. Model Comparison

We carried out comparative experiments to validate whether combining the no-arbitrage futures pricing model with SF-Transformers can improve the accuracy of predicting stock index futures prices. To validate the effectiveness of Transformers, we compared SF-Transformers with mainstream machine learning models. Herein, two machine learning models were chosen for comparison, namely long short-term memory (LSTM) and multilayer perceptron (MLP). Besides the above machine learning models, we compared the proposed method with an econometric model, i.e., the stock index futures arbitrage-free pricing model, as described in Section 2.1.

The LSTM, a recurrent neural network architecture, is specifically engineered to address prolonged dependencies and sequence prediction tasks. It achieves this by incorporating memory cells capable of selectively retaining and updating information over extended durations, enabling the network to discern and memorize patterns within sequential data. In this study, we tailored a specialized variant of LSTM, denoted as SF-LSTM, for a comprehensive comparative analysis. The SF-LSTM is configured to receive three variables representing spot-forward information and the stock index futures price, mirroring the input variables of the SF-Transformer. Employing an iterative approach like that in [70], SF-LSTM forecasts stock index prices with a forward-looking perspective, extending its predictive capabilities up to 28 days ahead.

The MLP, a feedforward neural network, comprises multiple fully-connected layers and proves effective for stock prediction tasks by learning to map input features to output values, such as stock prices, through a sequence of nonlinear transformations. In our configuration, the MLP neural network includes three fully-connected hidden layers. The input variables for the MLP mirror those of SF-Transformer and SF-LSTM, giving rise to the SF-MLP. The SF-MLP solely receives the variables from one day before the forecasting time horizons. Via multiple fully-connected layers, SF-MLP recurrently forecasts the stock index futures price and spot-forward model variables during the forecasting time horizons.

The stock index futures arbitrage-free pricing model takes the stock index price at initial time, risk-free interest rate, and stock index dividend yield as input, which are also included in the predictor variables of the SF-Transformer, SF-LSTM, and SF-MLP. In contrast to network models used by machine learning methods, the stock index futures arbitrage-free pricing model forecasts stock index futures at time horizons t using an analytic nonlinear model in Equation (5). In the following, we refer to the stock index futures arbitrage-free pricing model as the arbitrage-free model for brevity.

### 4.3. Ablation Study

This paper proposes the integration of spot-forward model variables into Transformer models to enhance overall model performance. To assess the significance of spot-forward parity model information in long-term forecasting, we conduct an ablation study on the SF-Transformer model. In this context, a baseline Transformer model is constructed, which only uses historical stock index futures prices for forecasting within the depicted time horizons in a generative manner, as illustrated in Figure 2.

To further explore the impact of spot-forward parity, we additionally conduct ablation studies on machine learning models, namely the LSTM and MLP. Both of these models rely solely on historical stock index futures prices as input, generating rolling forecasts for upcoming days within the designated forecasting time horizons.

### 4.4. Mutual Information Analysis

To evaluate the impact of the spot-forward parity model on forecasting stock index futures prices, we conduct a mutual information analysis. This involves calculating the mutual information between forecasted prices and predictor variables with and without spot-forward parity, as outlined in Section 2.5. We investigate the mutual information across various forecasting time horizons (1, 3, 5, 7, 10, 14, 21, and 28 days). The optimal predictors, with and without spot-forward parity, are determined by comparing the MAPEs obtained in the ablation analysis. We then utilize the variation of mutual information, as defined in Equation (23), to estimate the information gain from the inclusion of spot-forward parity variables.

## 5. Results and Discussion

**Figure 3:**
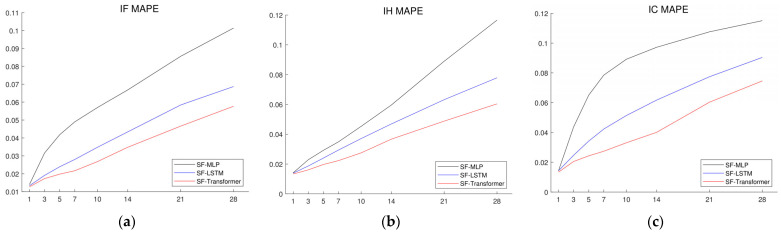
1-day- to 28-days-ahead out-of-sample forecasting errors of SF-Transformer, SF-LSTM, and SF-MLP for (**a**) IF, (**b**) IH, and (**c**) IC Stock Index Futures measured by MAPE. Note that arbitrage-free is not illustrated here due to its significantly higher and even inapplicable MAPEs.

Table 2 exhibits the results of the model comparison generated by the SF-Transformer, SF-LSTM, SF-MLP, and arbitrage-free pricing model for the CSI 300 Stock Index Futures (IF), SSE 50 Stock Index Futures (IH), and CSI 500 Stock Index Futures (IC) spanning the 1st to the 28th day. Figure 3 compares the MAPEs of three machine learning models—SF-Transformer, SF-LSTM, and SF-MLP—over 28 forecasting horizons in detail, either for CSI 300 Stock Index Futures, CSI 500 Stock Index Futures, or SSE 50 Stock Index Futures. The predictive performance of the SF-Transformer model is superior to those of SF-LSTM and SF-MLP. This suggests that the Transformer model can enhance the prediction accuracy. Compared to the SF-MLP that only receives information in the previous time point, the SF-Transformer and SF-LSTM take the long time series as input and find their interconnection to values of prediction time horizons. In contrast to LSTM that receives the input variables recurrently, the Transformer analyzes the entire time series concurrently, leveraging its attention mechanism to discern intricate dependencies and capture long-range relationships, resulting in a more holistic understanding of sequential data. The arbitrage-free model yields the highest MAPEs among the comparative models. For forecasting IF and IC, the MAPE values are even higher than the ones at the mid-term and long-term horizons. This indicates that, on average, the forecasted prices deviate from the actual prices by more than 100%, resulting in inapplicable forecasting results. The high deviation between the futures market prices and the theoretical prices forecasted by the arbitrage-free model is due to its rigorous theoretical assumptions. In comparison, machine learning models, especially the SF-Transformer, which learns the historical variation features of stock index futures prices and spot-forward parity variables, can avoid the limitations of these theoretical assumptions while incorporating the indicative factors within spot-forward parity to achieve significantly enhanced forecasting accuracy.

**Figure 4:**
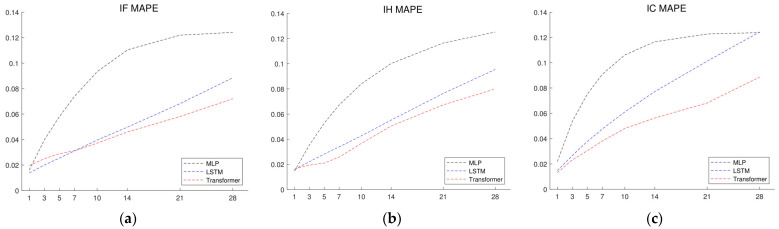
1-day- to 28-days-ahead out-of-sample forecasting errors of Transformer, LSTM, and MLP for (**a**) IF, (**b**) IH, and (**c**) IC Stock Index Futures measured by MAPE.

Figure 4 and Table 3 present the results of the ablation study, which is derived from vanilla Transformer, LSTM, and MLP models. Notably, the MAPEs obtained from the vanilla Transformer, LSTM, and MLP are consistently higher across all forecast horizons compared to their respective versions integrated with the spot-forward model (i.e., SF-Transformer, SF-LSTM, and SF-MLP). This disparity demonstrates the crucial role played by the integration of the spot-forward model in augmenting predictive performance. The findings suggest a critical divergence from data-driven machine learning models that rely only on historical price data. SF-Transformer, SF-LSTM, and SF-MLP benefit from the incorporation of the spot-forward parity, which is proven to be essential in capturing intricate financial nuances. This fusion of economic principles and financial information, rooted in the no-arbitrage futures pricing model, contributes to a more robust and informed forecasting model for stock index futures prices.

Of particular note is the observation that the MAPEs for the short- and mid-term days generated by the vanilla Transformer are notably worse than those produced by the vanilla LSTM. This indicates a clear performance degradation when relying solely on historical price data. The performance degradation may be attributed to the dependence on short-term stock futures prices in recent days, while Transformer models adopt a holistic time sequential approach but involve simple price to price attention values for predictions. In contrast, SF-Transformers leverage spot-forward model variables to generate in-depth self-attention values correlated with stock index futures prices. This self-attention mechanism enables the Transformer model to discern relevant time points, facilitating accurate predictions across both short- and long-term forecast horizons.

**Figure 5:**
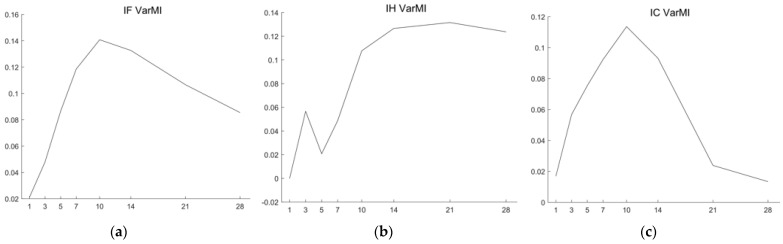
Variation of mutual information (VarMI) with and without spot-forward parity across forecasting horizons from 1 to 28 days for (**a**) IF, (**b**) IH, and (**c**) IC Stock Index Futures.

Figure 5 and Table 4 illustrate the variations in mutual information between forecasting prices and predictor variables with and without spot-forward parity. Utilizing the optimal predictors identified at each forecasting horizon (as detailed in Table 2 and Table 3), we compute the mutual information. Given that the SF-Transformer emerges as the optimal predictor for most forecasting horizons across all stock index futures (except for the first day in IC forecasting), the variations in mutual information serve as representative indicators of the information gain from spot-forward parity via the Transformer model. Notably, all variations in mutual information are positive except for the first day in IH forecasting, indicating a positive information gain from spot-forward parity in forecasting stock index futures prices. Particularly noteworthy is the relatively higher increase in mutual information observed at long-term forecasting horizons (H ≥ 10) compared to short-term ones (H < 10), suggesting the effectiveness of spot-forward parity in supporting long-term forecasting and reducing forecasting uncertainty.

For IH forecasting, the highest information gain is achieved in long-term forecasting horizons from H = 14 to H = 28 days. Conversely, for IF and IC forecasting, the information gain generally increases gradually from H = 1 to H = 10 days, with a peak increase in mutual information at the H = 10 forecasting day, followed by a decrease, especially during forecasting horizons from H = 21 to H = 28 days. Given that IH corresponds to the SSE 50 Index, a blue-chip index known for stable financial performance, it is reasonable to infer that spot-forward parity is informative for long-term forecasting of stock index futures prices. In contrast, IC, corresponding to the CSI 500, which reflects 500 middle and small stocks with good liquidity, exhibits larger price fluctuations and higher volatility, making spot-forward parity more effective in forecasting horizons from H = 7 to H = 14. As for IF, corresponding to the CSI 300 index comprising the top 300 blue-chip and GEM stocks, it combines characteristics of large-cap focus and high market sensitivity. Hence, the variation of mutual information for IF combines the characteristics of IH and IC, showing high information gain in forecasting horizons from H = 7 to H = 14 days, while still maintaining a median information gain in forecasting horizons up to H = 28 days. Overall, the variation in mutual information is consistent with the ablation analysis, highlighting the crucial role of spot-forward parity in enhancing stock index futures price forecasting, particularly in long-term forecasting scenarios.

While machine learning models demonstrate their strengths, as shown in Table 2, Table 3 and Table 4 and Figure 3, Figure 4 and Figure 5, it is important to consider the limitations of deep learning models. Firstly, deep learning models are data-driven and require substantial historical sequential data for training. When historical data are sparse or incomplete, the performance of these models can be severely impacted. Moreover, deep learning models can be sensitive to small changes or noise in the input data and may not perform well on data that differ significantly from the training data. This sensitivity is inherent to the data-driven nature of machine learning, where the model relies solely on the available data. On the other hand, the arbitrage-free model, an econometric model, is based on stock index futures pricing theory and determines fair values of stock index futures without historical data. However, econometric pricing models depend on strong assumptions, such as the no-arbitrage condition, and is a form of ideal analytic expression, which may not hold in real markets.

Therefore, for long-term forecasting that includes “unseen” variations in historical data, our SF-Transformer model improves machine learning performance by incorporating the econometric pricing model. This approach leverages the power of deep learning models to quantify nonlinear relationships within the data and utilizes associations provided by econometric theory to mitigate uncertainty in long-term forecasting. As shown in the mutual information analysis in Figure 5, the spot-forward parity provides valuable pricing information for long-term forecasting.

The performance of mutual information-enhanced deep learning models can be further improved by the use of Transformers. Compared with LSTM and MLP, this network architecture allows us to capture pricing-associated features from a holistic perspective of the historical spot-forward parity and price data while drawing attention to critical time intervals to effectively handle the complexity and uncertainty in mid-term and long-term forecasting of stock index price futures. However, due to large network parameters involved in the Transformer model, its performance can degrade significantly with small datasets, where simpler models such as MLP and LSTM might perform better.

Overall, by incorporating the economic principles and financial information embedded in the spot-forward parity futures pricing model, the SF-Transformer futures prediction model is considered reliable and applicable across various stock index futures markets in China. Since this model is also suitable for forecasting forward and commodity futures, it can be applied to predict prices in other forward and commodity futures markets, demonstrating strong generalization capabilities.

Based on the application results of the model presented in this research, the following recommendations are proposed for the policymakers, as well as for enterprises and investors:

1. Adopt mutual information-enhanced forecasting model: Consider integration of financial theory into deep learning model to leverage both economic principles and financial prices. This allows the mutual information generated by financial theory to reduce uncertainty in long-term forecasting and thus substantially improve predictive accuracy across different stock index futures.
2. Longer-term forecasting: Given the model’s capability to provide reliable forecasts even for longer-term periods, financial risk regulatory agencies and market participants may benefit from incorporating such forecasts into their risk management strategies. This is particularly relevant for entities engaged in long-term hedging using futures contracts.

## 6. Conclusions

This paper presents an integrated framework of financial theory and state-of-the-art deep learning methods, leveraging the respective strengths of both approaches to enhance long-term forecasting accuracy of stock index futures prices. The proposed SF-Transformer model, which combines spot-forward parity with the Transformer architecture, represents a significant advancement. By leveraging the self-attention mechanism of the Transformer, the SF-Transformer is capable of exploring the relationships between predictor variables, particularly those influenced by spot-forward parity, enabling it to learn intricate associations for forecasting prices. The comparative analysis of the experiments conducted in this research demonstrates that the SF-Transformer model surpasses other models in its predictive accuracy for major stock index futures prices in China.

As evidenced by enhanced mutual information, the spot-forward parity results in a substantial reduction in uncertainty in long-term forecasting. This highlights the crucial roles of stock index futures price, risk-free rate, and stock index dividend yield in long-term Chinese Stock Index Futures price forecasting. Furthermore, it suggests that information derived from both the spot market and the financial market is indispensable for the accurate forecasting of stock index futures prices.

Thanks to the enhanced mutual information, the SF-Transformer demonstrates robust performance, particularly in its long-term forecasting capability, allowing us to cover the entire lifecycle of the main contracts effectively. This extended forecasting horizon is of paramount importance for enterprises and investors involved in long-term hedging and risk management using stock index futures. The superior forecasting accuracy attained by SF-Transformer model positions it as a valuable tool for anticipating market trends and making informed decisions in the context of complexity in financial markets.

Serving as a viable forecasting solution, the SF-Transformer model contributes to the broader objective of creating more robust and comprehensive approaches against complexity and uncertainties in financial markets. In the realm of financial regulatory oversight, bolstering the pricing system of stock index futures emerges as a pivotal strategy for fostering stability in the Chinese Stock Index Futures market. To achieve this, it is imperative for regulatory authorities to factor in the influence of financial components such as the spot stock price, dividend yield, and the risk-free rate during monitoring activities. Instituting regulatory measures that encompass both short-term and long-term perspectives becomes crucial for preemptively identifying potential price fluctuations and implementing corresponding preventive actions. For enterprises and investors, the integration of financial theories with advanced deep learning models presents an avenue for developing a more robust forecasting model. This integration will enable them to uncover critical insights essential for navigating the complexity of stock index futures and other commodity markets, thereby making more informed decisions and mitigating risks effectively.

In the future, we will carry out trading simulations of stock index futures prices to further validate the effectiveness of the SF-Transformer. Metrics such as alpha and Sharpe ratios can be used to assess the investment return and effectiveness of the investment strategy. A trading simulation over a one-week, one-month, and two-month ahead forecast would fully validate the effectiveness and robustness of the SF-Transformer in real time market conditions. This would, in addition to the accuracy validation reported in this paper, provide a comprehensive evaluation of the model’s real-world applicability and robustness in complicated market conditions.

## Figures and Tables

**Table 1:** Summary of descriptive statistics.

Variables	Mean	Std. Dev.	Min.	Max.	Skew.	Kurt.
**IF**						
F	3970.9668	655.4405	2749.6000	5801.0000	0.5308	2.3216
S	3989.3080	647.9869	2853.7600	5807.7200	0.5447	2.3349
q	2.2462	0.2600	1.4701	2.9306	0.1938	3.1360
*r_f_*	2.0884	0.5122	0.6755	3.4819	−0.4059	2.5593
ln*F*	8.2736	0.1615	7.9192	8.6658	0.2777	2.1369
ln*S*	8.2786	0.1588	7.9564	8.6669	0.2974	2.1368
**IH**						
F	2775.3841	435.6221	1809.8000	4020.4000	0.2494	2.4051
S	2782.7353	430.5829	1912.7200	4028.5300	0.2713	2.4193
q	2.9325	0.3354	1.5295	4.0738	0.1671	3.2885
*r_f_*	2.0884	0.5122	0.6755	3.4819	−0.4059	2.5593
ln*F*	7.9162	0.1575	7.5010	8.2991	−0.0556	2.2782
ln*S*	7.9192	0.1549	7.5563	8.3012	−0.0270	2.2594
**IC**						
F	6103.2134	984.4297	4033.2000	11,427.8000	1.1983	7.9357
S	6159.7779	1004.0954	4018.4600	11,545.8900	1.1701	7.5655
q	1.1098	0.3869	0.3647	2.1044	0.2407	2.2403
*r_f_*	2.0884	0.5122	0.6755	3.4819	−0.4059	2.5593
ln*F*	8.7043	0.1556	8.3023	9.3438	0.2172	4.5639
ln*S*	8.7132	0.1573	8.2987	9.3541	0.2147	4.4870

Note: This table reports the main descriptive statistics of the variables under consideration over the whole sample period from 16 April 2015 to 25 October 2022. The main descriptive statistics include the mean, standard deviation, minimum, maximum, skewness, and kurtosis.

**Table 2:** 1-day- to 28-days-ahead out-of-sample forecasting errors of SF-MLP, SF-LSTM, SF-Transformer, and arbitrage-free models for IF, IH, and IC Stock Index Futures measured by MAPE. N/A represents the forecasting result is not applicable, with MAPEs > 1.

Models	H = 1	H = 3	H = 5	H = 7	H = 10	H = 14	H = 21	H = 28
**IF**								
SF-MLP	0.0140	0.0316	0.0418	0.0490	0.0570	0.0668	0.0856	0.1014
SF-LSTM	0.0133	0.0191	0.0239	0.0280	0.0348	0.0434	0.0584	0.0687
SF-Transformer	0.0127	0.0173	0.0198	0.0217	0.0268	0.0348	0.0466	0.0577
Arbitrage-free	0.3361	0.6810	N/A	N/A	N/A	N/A	N/A	N/A
**IH**								
SF-MLP	0.0146	0.0353	0.0528	0.0672	0.0841	0.1002	0.1165	0.1252
SF-LSTM	0.0160	0.0221	0.0282	0.0339	0.0427	0.0552	0.0764	0.0955
SF-Transformer	0.0162	0.0195	0.0210	0.0258	0.0367	0.0505	0.0673	0.0800
Arbitrage-free	0.5765	0.8629	0.9387	0.9665	0.9820	0.9887	0.9929	0.9944
**IC**								
SF-MLP	0.0143	0.0437	0.065	0.0785	0.0892	0.0972	0.1076	0.1151
SF-LSTM	0.0143	0.0247	0.0342	0.0423	0.0514	0.0617	0.0774	0.0904
SF-Transformer	0.0134	0.0205	0.0243	0.0274	0.0331	0.0401	0.0603	0.0746
Arbitrage-free	0.7147	N/A	N/A	N/A	N/A	N/A	N/A	N/A

**Table 3:** 1-day- to 28-days-ahead out-of-sample forecasting errors of MLP, LSTM, and Transformer forecasting models for IF, IH, and IC Stock Index Futures measured by MAPE.

Models	H = 1	H = 3	H = 5	H = 7	H = 10	H = 14	H = 21	H = 28
**IF**								
MLP	0.0161	0.0398	0.0581	0.0739	0.0933	0.1103	0.1219	0.1241
LSTM	0.0138	0.0200	0.0255	0.0311	0.0396	0.0497	0.0682	0.0885
Transformer	0.0196	0.0250	0.0289	0.0313	0.0372	0.0459	0.0581	0.0720
**IH**								
MLP	0.0141	0.0229	0.0292	0.0349	0.0453	0.0596	0.0890	0.1166
LSTM	0.0139	0.0187	0.0240	0.0294	0.0371	0.0469	0.0632	0.0779
Transformer	0.0133	0.0161	0.0197	0.0223	0.0275	0.0367	0.0488	0.0604
**IC**								
MLP	0.0220	0.0538	0.0752	0.0909	0.1062	0.1165	0.1227	0.1238
LSTM	0.0148	0.0267	0.0377	0.0476	0.0611	0.0771	0.1013	0.1243
Transformer	0.0132	0.0233	0.0304	0.0381	0.0480	0.0563	0.0682	0.0886

**Table 4:** Summary of mutual information variation with and without spot-forward parity across forecasting horizons from 1 to 28 days for IF, IH, and IC Stock Index Futures.

Models	H = 1	H = 3	H = 5	H = 7	H = 10	H = 14	H = 21	H = 28
**IF**	0.0211	0.0480	0.0866	0.1185	0.1408	0.1327	0.1066	0.0854
**IH**	0.0000	0.0566	0.0208	0.0488	0.1077	0.1266	0.1316	0.1237
**IC**	0.0169	0.0568	0.0754	0.0923	0.1136	0.0931	0.0239	0.0134

## Data Availability

Data is contained within the article.
